# Journal for immunotherapy of cancer reviewer acknowledgement 2015

**DOI:** 10.1186/s40425-016-0113-5

**Published:** 2016-02-16

**Authors:** Pedro Romero

**Affiliations:** Ludwig Center for Cancer Research, University of Lausanne, Lausanne, Switzerland

## Abstract

On behalf of the JITC Section Editors and Associate Editors, I wish to acknowledge with sincere appreciation the assistance of the following reviewers who generously contributed their time and efforts in 2015 in the appraisals of manuscripts submitted to the journal. These reviewers not only have been invaluable in assessing the merit of submitted studies but also, by their careful analysis and critique and their general and specific constructive recommendations, have often greatly enhanced the value of these manuscripts. The quality of JITC can be attributed in large measure to their dedication and we are sincerely grateful.

**Carlos Alfaro**

Spain

**Ramon Arens**

Netherlands

**Shailender Bhatia**

USA

**Christian Blank**

Netherlands

**Marie Bleakley**

USA

**Renier Brentjens**

USA

**Martin Cannon**

USA

**Paola Cappello**

Italy

**John Castle**

Germany

**Ugo Cavallaro**

Italy

**Bartosz Chmielowski**

USA

**Joe Clark**

USA

**Alice Conlin**

USA

**Bruno Correia**

USA

**Todd Crocenzi**

USA

**Brendan Curti**

USA

**Toos Daemen**

Netherlands

**Patrick Dillon**

USA

**Daniela Dinulescu**

USA

**Reinhard Dummer**

Switzerland

**Janice Dutcher**

USA

**Lea Eisenbach**

Israel

**Zhichao Fan**

USA

**Mark Faries**

USA

**Morganna Freeman-Keller**

USA

**Claus Garbe**

Germany

**Ulrike Gnad-Vogt**

Germany

**John Godwin**

USA

**Gullu Gorgun**

USA

**John Haanen**

Netherlands

**M Heemskerk**

Netherlands

**Peter Hersey**

Australia

**Rafat Husain**

USA

**Theodore Johnson**

USA

**Harriet Kluger**

USA

**Viktor Koelzer**

Switzerland

**Holbrook Kohrt**

USA

**Lawrence Lamb**

USA

**Heinz Läubli**

Switzerland

**Peter Lee**

USA

**Qiao Li**

USA

**Devin Lowe**

USA

**Jason Luke**

USA

**Jose Lutzky**

USA

**Giuseppe Masucci**

Sweden

**Jeffrey Miller**

USA

**Christian Munz**

Switzerland

**Thorunn Olafsdottir**

Sweden

**Eileen O'Reilly**

USA

**Patrick Ott**

USA

**David Page**

USA

**Julian Pardo**

Spain

**Manish Patankar**

USA

**Sapna Patel**

USA

**Jose Luis Perez Gracia**

Spain

**Shari Pilon-Thomas**

USA

**John Powderly**

USA

**Daniel Powell**

USA

**Ryan Sullivan**

USA

**Paul Tumeh**

USA

**Dominique Vanhecke**

Switzerland

**John Vetto**

USA

**Michael Von Bergwelt**

Germany

**Derek Wainwright**

USA

**Hassane Zarour**

USA

**Jacques Zimmer**

Luxembourg

